# Crisis readiness for rare disease populations: learnings and recommendations by the European Reference Networks

**DOI:** 10.1016/j.lanepe.2026.101801

**Published:** 2026-08-08

**Authors:** Hélène Dollfus, Alexis Arzimanoglou, Teresinha Evangelista, Holm Graessner, Marta Mosca, Luca Sangiorgi, Jean-Yves Blay, Christine Bodemer, Pierre Fenaux, Francisco Hernández, Guillaume Jondeau, Marjolijn J.L. Ligtenberg, Ansgar W. Lohse, Irene M.J. Mathijssen, Peter Mulders, Alberto M. Pereira, Franz Schaefer, Joost F. Swart, Alain Verloes, Thomas O.F. Wagner, René M.H. Wijnen, Arthur A.M. Wilde, María del Mar Mañú Pereira, Ruth Ladenstein, Maurizio Scarpa

**Affiliations:** a ERN-EYE, FSMR SENSGENE, Centre de Référence pour les Affections Rares en Génétique Ophtalmologique (CARGO), IGMA, Hôpitaux Universitaires de Strasbourg, Université de Strasbourg, Strasbourg, France; b ERN EpiCARE Coordinator, Child Neurology and Epilepsy Department, Children's University Hospital San Juan de Dios and IRSJD, Barcelona, Spain; c ERN EURO-NMD, Department of Neuropathology, Nord/Est/Ile-de-France Neuromuscular Reference Center, Pitié-Salpêtrière Hospital, APHP, Sorbonne University, Paris, France; d ERN-RND, University Hospital Tübingen, Institute for Medical Genetics and Applied Genomics and Center for Rare Diseases, Tübingen, Germany; e ERN ReCONNET, Rheumatology Unit, Azienda Ospedaliero-Universitaria Pisana, Department of Clinical and Experimental Medicine, University of Pisa, Pisa, Italy; f ERN BOND, Department of Rare Skeletal Disorders, IRCCS Istituto Ortopedico Rizzoli, Bologna, Italy; g ERN EURACAN, Centre Léon Bérard, & Université Claude Bernard Lyon I, Lyon, France; h ERN-Skin, Department of Dermatology, Expert Centre for rare Skin Disorders, Necker-Enfants Malades Hospital, APHP, Paris-Cité University, Paris, France; i ERN-EuroBloodNet, Service Hématologie Seniors, Hôpital Saint-Louis, Assistance Publique-Hôpitaux de Paris and Université Paris Cité, Paris, France; j ERN TransplantChild, Department of Pediatric Surgery, La Paz University Hospital, Madrid, Spain; k ERN VASCERN, Reference Center for Marfan Syndrome and Related Diseases, Cardiology Department, APHP, Hospital Bichat, Université Paris Cité, INSERM, Paris, France; l ERN GENTURIS, Department of Human Genetics, Department of Pathology, Research Institute for Medical Innovation, Radboud University Medical Center, Nijmegen, the Netherlands; m ERN RARE-LIVER, I. Department of Medicine, University Medical Center Hamburg-Eppendorf, Hamburg, Germany; n ERN CRANIO, Department of Plastic and Reconstructive Surgery and Hand Surgery, Erasmus University Medical Center Rotterdam, the Netherlands; o ERN eUROGEN, Department of Urology, Radboud University Medical Center, Nijmegen, the Netherlands; p Endo-ERN, Department of Endocrinology and Metabolism, Amsterdam University Medical Center, Amsterdam, the Netherlands; q ERN ERKNet, Division of Pediatric Nephrology, Department of Pediatrics, University of Heidelberg, Heidelberg, Germany; r ERN RITA, Department of Pediatric Immunology and Rheumatology, Wilhelmina Children's Hospital/UMC Utrecht, Utrecht, the Netherlands; s ERN ITHACA, Department of Genetics, APHP-Robert DEBRE University Hospital, Université Paris Cité Medical School, Paris, France; t ERN-LUNG, Frankfurt Reference Center for Rare Diseases (FRZSE), University Hospital Frankfurt, Goethe University Frankfurt, Frankfurt am Main, Germany; u ERN ERNICA, Department of Pediatric Surgery, Erasmus MC Sophia, Rotterdam, the Netherlands; v ERN GUARD-Heart, Department of Clinical Cardiology, Amsterdam University Medical Centre (location AMC), Amsterdam, the Netherlands; w ERN-EuroBloodNet, Hematology Research Group, Vall D'Hebron Research Institute, Barcelona, Spain; x ERN PaedCan Coordinator and Chair Coordinator's Group, St Anna Children's Hospital and Children's Cancer Research Institute, Medical University of Vienna, Vienna, Austria; y MetabERN Coordinator, Regional Coordinating Centre for Rare Diseases, University Hospital Udine, Udine, Italy; z Transplant Research Group, Institute for Health Research IdiPaz, Madrid, Spain

**Keywords:** Rare diseases, European Reference Networks, Health system resilience, Climate change, Crisis preparedness, Cross-border healthcare, Continuity of care, Health emergency response, Pandemics, Healthcare pathways

## Abstract

Patients with rare diseases are particularly vulnerable during emergencies due to dependence on specialised care pathways, medicines, and expertise. European Reference Networks (ERNs) for rare and complex diseases have operated since 2017 and were confronted with two major crises: the COVID-19 pandemic and the war in Ukraine. To assess ERN experiences, responses, and preparedness for crisis and disaster situations, we conducted a cross-sectional survey of all 24 ERN coordinators between July and September 2025. Only 2 (8·3%) ERNs reported pre-existing preparedness plans, while 70% lack structured frameworks. Major bottlenecks included patient access to healthcare facilities, drug and device shortages, bed shortage, staff unavailability, and diagnostic service interruption. Our findings highlight the need to formally integrate and mandate ERNs into European health emergency preparedness and response mechanisms. We propose a 10-point global crisis preparedness plan for rare diseases emphasizing cross-border coordination, digital infrastructure, patient education, and integration with national emergency services and non-governmental organizations.

## Introduction

Patients with rare and complex diseases represent among the most vulnerable populations during crises and disaster situations.^1^ Events at any scale—local, regional, national, or international—can rapidly undermine the foundations upon which these individuals depend for their clinical status, quality of life and even survival.^2^ Unlike common conditions, where care can be distributed across general healthcare systems, rare diseases require highly specialised expertise, essential medications with limited suppliers, specific medical devices, and uninterrupted care pathways that are complex, and consequently, exceptionally susceptible to disruption.

Crisis and disaster situations are increasing in frequency and intensity globally, driven by climate change, geopolitical instability, pandemic threats, and technological vulnerabilities including cyberattacks.^3^^,^^4^ The recently announced withdrawal from WHO of the United States, one of its founding members,^5^ will inevitably make the world less safe. Within this context, the European Health Union initiative has emphasized the need for strengthened health security coordination across Member States.^6^

The 24 European Reference Networks (ERNs) for rare and complex diseases were established under the Cross-border Healthcare Directive (2011/24/EU) and became operational in 2017.^7^^,^^8^ These networks, represented by 24 coordinators, now connect 1606 highly specialised clinical teams (known as healthcare providers (HCPs)) centres located in 375 hospitals across all EU Member States and Norway, representing a large population of healthcare professionals, providing expert care for an estimated 30 million Europeans living with rare diseases.^9^ Since their inception, ERNs have unexpectedly confronted two major crises: the COVID-19 global pandemic and the armed conflict following Russia's invasion of Ukraine in February 2022.

## Scope of the paper

Although relatively young networks, ERNs demonstrated remarkable responsiveness during both the COVID-19 pandemic and the Ukraine crisis with treatment alternatives in case of shortage of specific medications as well as generating vaccination guidance and educational webinars,^10^, ^11^, ^12^, ^13^ facilitating cross-border care, and coordinating support for displaced patients.^14^^,^^15^ While these experiences provided valuable lessons for future preparedness, the specific role of ERNs in crisis preparedness and response was not previously formally defined or systematically assessed. We therefore conducted a comprehensive survey of all 24 ERN coordinators as representants of their healthcare professionals to assess their experiences, responses, challenges, and recommendations for crisis and disaster preparedness, with the aim of informing European health policy and establishing ERNs as essential infrastructure for protecting vulnerable rare disease populations during critical situations based on ten recommendations for a preparedness plan.

## Capturing the experience of European Reference Networks

This study was conducted within the framework of the Joint Action JARDIN project (https://jardin-ern.eu/) co-funded by the European Union, which aims to support the integration of ERNs into national healthcare systems. We conducted a cross-sectional online survey involving all 24 ERN coordinators between July 1st and September 30th, 2025. The questionnaire was developed through an iterative process. First, a preparatory online meeting with patient representatives (April 1st 2025) was held to gather qualitative insights in addition to the coordinators reports. Based on these insights, clinicians’ experience, and methodological input from JARDIN Work Package 6, ERN-EYE drafted the initial survey, which was reviewed by ERN EpiCARE and MetabERN to ensure clinical relevance and applicability across ERNs. The questionnaire was then tested by skilled individuals not involved in the study to assess clarity and comprehensibility, and revised accordingly. The EUSurvey platform (https://ec.europa.eu/eusurvey/), a secure European Commission tool for institutional surveys, was then used for data collection.

The survey comprised 93 questions across six thematic domains: general crisis considerations, COVID-19 pandemic experience, Ukraine war response, lessons learned in ERNs preparedness, patients and professionals preparedness, and future recommendations (available in Supplemental Material 1). Question formats included dichotomous (yes/no, 56%, 53/93), single-choice (7%, 7/93), multiple-choice with multiple answers permitted (14%, 13/93), rating scales (8%, 8/93), ranking questions (5%, 5/93), and open-ended free-text responses (7%, 7/93). Average completion time was 30 min (range 20–60 min). All ERN coordinators provided informed consent for data use and publication, and no patient data was used. Statistical analysis was descriptive and based on frequencies and proportions calculated through EUSurvey's integrated data analysis tool. Dichotomous, single-choice, multiple-choice responses and rating questions were summarized as percentages, and ranking questions were analysed via contingency tables (rank, answer). Free-text responses were reviewed qualitatively to summarize the main concerns, provide clarification or illustrative examples but were not formally analysed in the present study for an extensive report.

All 24 ERN coordinators completed the survey (100% response rate) in order to deliver each network's experiences. Respondents represented ERN coordinating centres based in six EU Member States: Netherlands (n = 7), France (n = 6), Germany (n = 4), Italy (n = 3), Spain (n = 3), and Austria (n = 1). Collectively, the ERNs cover the full spectrum of rare and complex diseases including rare cancers, rare neurological and neuromuscular diseases, rare sensorial diseases, rare metabolic disorders, rare inflammatory and immune disorders, rare developmental diseases, rare pulmonary and cardiovascular diseases, rare skin diseases, rare kidney diseases, complex rare transplantation situations, rare liver diseases, rare endocrine diseases, and skeletal diseases. The summary of the results presented in this article can be found in Table 1.

**Table 1:** Summary of key survey findings across all 24 European Reference Networks.

Domain	n/N	%
COVID-19 pandemic		
Significant impact on ERN management/activity	22/24	91·6
Ukraine war response		
Immediate response to support Ukrainian patients	20/24	83·3
Contacts established in border countries for transfers	13/24	54·2
Efficient contact with Ohmatdyt Ukrainian hub	16/24	66·7
Crisis preparedness		
No pre-existing crisis plan before COVID-19	22/24	91·6
Currently lack effective preparedness plan	17/24	70·8
Cross-border care		
Anticipate urgent cross-border transfers needed	20/24	83·3
Consider genetic testing crucial during crises	16/24	66·7
Patient preparedness		
No patient preparedness plan exists	15/24	62·5
No emergency partners identified for training	15/24	62·5
Climate change		
Identified specific climate change threats to patients	9/24	37·5

## The European Reference Network experience during recent crises

### Assessing threats to rare disease care

**Fig. 1:**
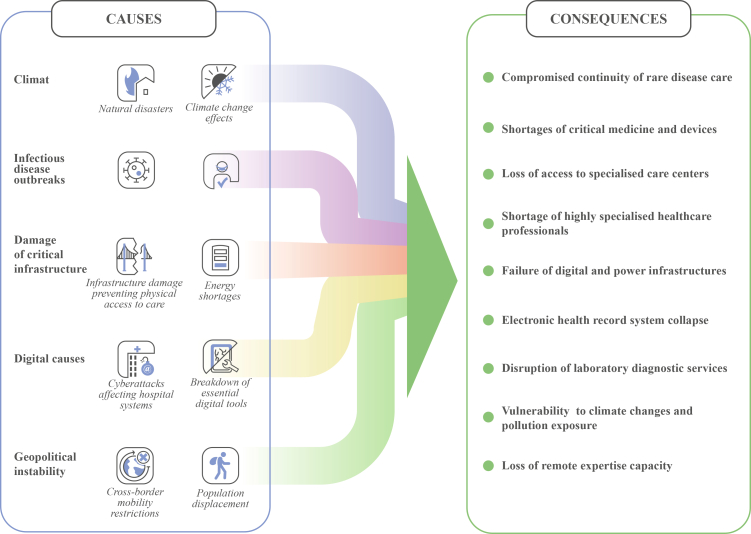
**Synoptic view of crisis causes and consequences for rare disease populations**. Causes and consequences are closely interconnected, with a single crisis driver potentially affecting multiple domains simultaneously, while consequences can amplify one another. Funding constraints represent a major threat, as they may arise from, and further worsen, nearly all aspects of crisis response and continuity of care.

Beyond COVID-19 and the Ukraine conflict, coordinators identified multiple threats that could critically impact rare disease care pathways. These included natural disasters (earthquakes, floods, wildfires), climate change effects (extreme heat and cold), infectious disease outbreaks, infrastructure damage preventing physical access to care, cyberattacks affecting hospital systems or ERN infrastructures, breakdown of digital tools essential to operations, major energy shortages, political instability, medical supply chain disruptions, cross-border mobility restrictions, and population displacement (See Fig. 1). Nine of 24 ERNs (37·5%) identified climate change as a specific threat to their patient populations. For example, rare cardiac diseases, immune diseases, endocrine disorders, pulmonary conditions,^16^ neuromuscular diseases,^17^ metabolic disorders, kidney diseases, and sickle cell disease are particularly vulnerable to thermoregulatory stress. Conditions sensitive to thermoregulatory stress, such as neuromuscular diseases with impaired temperature regulation, endocrine disorders affecting homeostasis, kidney diseases in which polyuria predisposes patients to dehydration and subsequent deterioration of kidney function, and sickle cell disease with vaso-occlusive crises triggered by temperature extremes, require targeted preparedness strategies.^16^ Similarly, high pollution levels have been associated with increased risk of cardiovascular diseases,^18^ or bone marrow failure syndromes and haematological malignancies.^19^

### Lessons from the COVID-19 pandemic

Twenty-two of 24 ERNs (91·6%) reported that COVID-19 significantly impacted their management and network activities, with substantial variation across countries. Some countries maintained near-normal paediatric surgical care due to low paediatric COVID-19 cases, while others experienced near-complete shutdown as staff were redeployed to COVID-19 wards.^20^^,^^21^ The primary challenge identified was discontinuity in care pathways, including loss of access to healthcare professionals, missed diagnostic or follow-up visits, and cancelled hospitalizations.^22^ Adaptation varied widely across Member States, particularly regarding telemedicine implementation, continuity of treatments, hospital access, and resource allocation.

As early as February 2021, all ERN coordinators collaborated to produce joint vaccination recommendations for rare disease patients, disseminated through patient associations, learned societies, and direct contacts with the European Commission (EC) and national health ministries.^14^ In parallel, ERNs developed their digital platforms (i.e. websites, social media …) to support diffusion of information to patients, families and experts, while the EC Clinical Patient Management System (CPMS) helped to support clinician exchange.^23^ Building on these expanded digital infrastructures, ERNs produced a wide range of COVID-19-related resources, including disease-specific guidance,^24^^,^^25^ educational webinars, patient communication materials, and COVID-19-specific registries.^26^^,^^27^ A complete overview of these documents is provided in Supplemental Table S1. ERNs also contributed to the identification of new clinical entities, such as Multisystem Inflammatory Syndrome in Children (MIS-C), leading to the creation of dedicated ERN registries such as HyperPed-Covid.^27^

### Lessons from the war in Ukraine

Within the first months following Russia's invasion on February 24th, 2022, 20 of 24 ERNs (83·3%) reported immediate responses to support Ukrainian patients and healthcare professionals, with significant impact on management team activities. Communication and information efforts intensified across 20 ERNs (83·3%), and 80% reported augmented activity in HCPs located in EU border countries.^28^

Initial collective actions included a joint support letter signed by all 24 coordinators and launch of the #ERNcare4Ua campaign to assist displaced Ukrainian patients with rare diseases.^15^ All 24 ERNs, through the Coordinators Chairs group, immediately established a weekly “Ukraine crisis call for Rare Diseases” with representatives of EC and DG SANTE, the French Presidency of the Council and European Parliament representatives. They were also regularly in contact with the European Medical Agency, EURORDIS and several patient associations, the Red Cross, the WHO and several NGOs.

Thirteen ERNs (55%) established contacts in border countries for patient transfers, and ten (42%) used CPMS for online medical consultations. ERNs liaised with Ukrainian hospitals, global and national patient organizations, Member State authorities, national contact points, Red Cross, and ministries of health.^29^

In December 2022, a central Ukrainian hub for rare diseases was established at Ohmatdyt Children's Hospital in Kyiv. Since 2025, this hub is financially supported by the EC via the aforementioned JARDIN project. 16 ERNs (66%) have maintained efficient contact, primarily for training, clinical collaborations, and CPMS discussions as the system was made accessible by the EC to Ukrainian colleagues, patient referrals, and drug support. Thirteen ERNs (55%) provided training in the form of short stays at European HCPs, and 55% translated communication materials into Ukrainian. A complete overview of these documents is provided in Supplemental Table S2. In addition to the over commitment of countries sharing borders with Ukraine, the three main ongoing challenges identified were disruption of care continuity, drug shortages, and training needs for Ukrainian professionals.

### Preparedness to face future crises

Twenty-two of 24 ERNs (91·6%) had no pre-existing crisis preparedness plans before COVID-19. Eleven ERNs (45%) developed plans during the crisis, but currently 17 (70%) still lack effective preparedness plans. ERNs self-evaluated their crisis effectiveness as only moderately satisfactory, though nearly all expressed willingness to pursue readiness efforts. Four critical bottlenecks were identified: patient accessibility to healthcare facilities (diagnosis, therapy, emergency); drug, device, and equipment shortages; staff unavailability and shortages; and interruption of diagnostic services. Examples of documents prepared by ERNs to prepare for or mitigate those issues is provided in Supplemental Table S3.

### Cross-border care planning

Twenty of 24 ERNs (83·3%) anticipate that urgent or semi-urgent cross-border physical transfer of patients may be required during future crisis. Critical scenarios include for example all types of cancer (paediatric and adult), urgent cardiac surgery (especially congenital heart disease), transplantation programs, dialysis, specialized intensive care for neuromuscular conditions, metabolic intensive care for intoxication syndromes, prolonged Video-EEG monitoring prior to epilepsy surgery, and neonatal or paediatric surgical emergencies. Cross-border channels are also essential to ensure continuity in the supply of medications, medical devices, laboratory consumables, and access to genetic testing services.

Sixteen ERNs (67%) considered genetic testing crucial during crisis, particularly for treatment eligibility decisions (e.g., gene therapy for Spinal Muscular Atrophy). Disruptions to routine laboratory services were reported to create substantial risks of diagnostic delays and missed therapeutic windows, especially for newborns or rapidly progressive diseases.

### Preparing and empowering patients

Fifteen of 24 ERNs (62·5%) currently have no patient preparedness plan, while only seven ERNs (30%) have directly involved patients in designing preparedness strategies. The most effective patient preparedness measures identified were reliable patient-provider communication channels, connection to local emergency services, remote patient monitoring capabilities, emergency medication supplies, and access to rescue medications, and patient self-care education. Priority topics for patient preparedness include emergency preparedness education materials, virtual support groups, training on treatment self-administration, and systems for storing and sharing emergency contact information. Certain patient groups may be particularly vulnerable in this context, including those transitioning from paediatric to adult care, a process that is already complex under routine conditions and may be further critically compromised and even missed during emergencies and crisis situations.

### Maintaining clinical trials during crises

Given the limited availability of approved therapies for many rare diseases, clinical trials often represent the only access to potentially lifesaving or disease-modifying treatments. Consequently, nine of 24 ERNs (37%) reported that interruption of clinical trials during crises could result in severe complications or life-threatening events such as irreversible loss of function or death, particularly in conditions such as neuromuscular disorders, rare metabolic diseases, developmental epileptic encephalopathies and rare cancers.

The main barriers to trial continuity included patients’ inability to attend in-person visits, redeployment of clinical staff to crisis response activities, limitations in laboratory and diagnostic services, site closures or access restrictions, and disruptions to investigational product supply chains. ERNs advocated for strengthened remote trial models, including tele-visits, home monitoring, and home delivery of investigational products, as well as pre-approved flexible protocols developed in collaboration with regulators and trial sponsors.

### Engaging emergency response partners

Fifteen of 24 ERNs (62·5%) have not identified emergency partners to receive rare disease training or awareness materials. For those that have, the most relevant partners identified were HCP emergency services staff, emergency medical services, and governmental emergency management services. ERN EURO-NMD reported plans to extend training to civil protection agencies and EU emergency frameworks, with documented positive outcomes including fewer treatment disruptions, lower hospitalization and mortality rates, and structural improvements in national preparedness plans.

## Drawing insights from ERN responses to recent crises

This first comprehensive survey of all 24 ERN coordinators reveals that ERNs demonstrated rapid response capacity during two distinct crises even in the absence of a coordinated preparedness plan but still lack systematic structured global preparedness frameworks. Despite being young networks at the time of COVID-19's emergence, ERNs spontaneously assumed multiple collective roles: generating clinical guidance, facilitating telemedicine,^7^^,^^22^^,^^30^^,^^31^ coordinating patient communication, and supporting cross-border care. These experiences highlight ERNs' potential as an essential crisis response infrastructure for rare disease populations within the European Health Union and the urge to secure funding to address the 10 points cited hereunder.

The Ukraine crisis demonstrated that ERNs can rapidly mobilize for geopolitical emergencies, not only pandemics. The #ERNcare4Ua initiative (https://www.erncare4ua.com), joint coordinator statements, and establishment of the Ohmatdyt National Specialized Children Hospital in Kyiv as a rare disease hub illustrate their collaborative capacity. However, this response was ad hoc rather than systematic. Formal integration of ERNs with humanitarian organizations such as Médecins Sans Frontières, WHO, Red Cross, and DG ECHO was not fully established, highlighting the need for pre-existing reinforced relationships and protocols.

Additional threats are identified such as climate change that is now emerging as an underrecognized threat, with over one-third of ERNs identifying specific vulnerabilities in their patient populations.

Although ensuring clinical trial continuity is not a formal mandate of ERNs, their clinical expertise and network structures position them well to support adaptive trial organisation during crises. Importantly, many of the barriers affecting trial continuity, such as diagnostic and laboratory limitations, staff redeployment, and supply chain disruptions, have already been identified by ERNs as challenges to the continuity of care for patients with rare diseases. As a result, the solutions, tools, and organisational structures being developed by ERNs to address these challenges in routine care could also be leveraged to support the continuity of clinical trials during crisis situations.

## ERNs operate in a large ecosystem

Our study has limitations. Survey responses reflect coordinators perspectives and may not capture all HCP experiences within the networks. Self-reported effectiveness assessments may be subject to recall bias and social desirability effects. In the future, in-depth patient impact could be quantified through the use of registry data. The study was conducted retrospectively, using an online survey facility with self-reported data that can induce a bias during a period of relative stability, and coordinator perceptions might differ during active crisis. Of note, the study recorded the ERN position by way of the coordinators, which may introduce a system-level perspective bias and does not capture variability across individual HCPs or patient experiences within networks.

Additionally, we focused on ERN-level responses and did not systematically assess integration with national emergency systems across all Member States. Our findings should be strengthened through dedicated future prospective studies enabling more comprehensive analyses, including subgroup and regional comparisons, and corroborated using external data sources such as patient registries or national indicators. Interestingly, our work points to the lack of existing dedicated external validation tools underpinning the importance of the field to be urgently addressed by national, European and international authorities. Moreover, ERNs already work in close symbiotic collaboration with patient associations that will lead to thorough future prospective studies.

## Developing a preparedness framework for rare disease populations

Still, the survey identifies four main bottlenecks—patient accessibility, supply shortages, staff unavailability, and diagnostic interruption—that align with broader health system vulnerabilities but are amplified for rare disease populations. Patients with rare diseases often depend on single-source medications, highly specialized equipment (i.e. dialysis, ventilators, tube feeding systems), and expertise concentrated in a limited number of centres.^32^ Disruption at any point in these fragile care pathways can rapidly become life-threatening. The areas affected by crises are also closely interdependent, with challenges in one domain rapidly affecting others, and all being highly sensitive to funding constraints, which may impact multiple components of care and research at once.^33^

Overall, our findings demonstrate significant gaps in preparedness despite proven response capacity. Over 90% of ERNs lacked pre-existing crisis plans, and 70% still lack effective plans. This paradox—high responsiveness but low preparedness—reflects the networks’ current status as primarily clinical and knowledge networks without formal crisis mandates or dedicated and sustainable resources. ERNs coordinate expert care under normal circumstances but are not procurement bodies, emergency services, or officially designated disaster response organizations.

## A 10-point set of urgent recommendations for rare diseases patients in the context of crisis and disaster

**Fig. 2:**
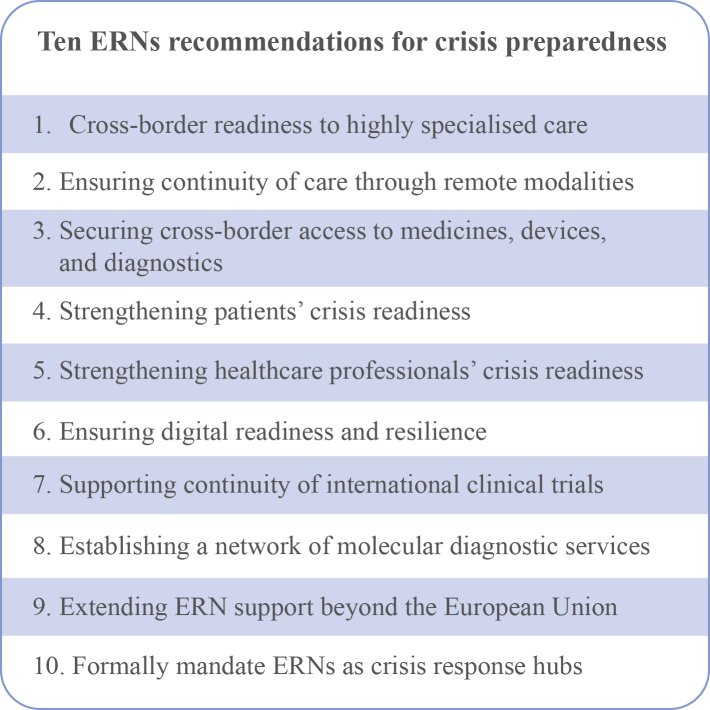
**ERNs' ten recommendations for Global Crisis Preparedness Plan towards the rare and complex diseases European population**.

Based on these findings, we propose in Panel 1 a 10-point set of recommendations for a European Global Preparedness Plan for people living with rare and complex diseases (summarised in Fig. 2). This plan underscores the need to mandate ERNs as central actors to anticipate and activate joint national and European crisis coordination hubs to maintain continuity of care.

Panel 1 **Detailed description of the ten ERN recommendations for a global crisis preparedness plan.**

**Recommendation n° 1: Cross border readiness to highly specialised care**

Ensuring patient physical continuous access to expert care centres requires pre-established patient transfer agreements between ERN centres to enable redistribution of care when facilities are overwhelmed, alongside simplified cross-border healthcare mechanisms allowing patients to access care at any ERN centre during crises affecting their home country. Cross-border readiness should also include pre-established agreements with surrogate laboratories and clinical trial sites to ensure continuity. Long-standing cooperative relationships between centres across member states, supported by twinning initiatives and joint educational programmes, facilitate rapid and effective implementations.

**Recommendation 2: Ensuring continuity of care through remote modalities**

When physical access is not possible, continuity of care should be ensured through strengthened remote modalities, including secure professional communication platforms, teleconsultation, and telemedicine enabling access to ERN expertise at national and EU levels.

**Recommendation 3: Securing cross-border access to medicines, devices, and diagnostics**

To address resource vulnerabilities, cross-border channels should be pre-established for essential medicines, innovative therapies, medical devices, laboratory consumables, and bioinformatics support for genomic interpretation. These channels should be supported by coordinated European supply chains, strategic stockpiling across multiple locations, and reduced dependency on single external suppliers.

**Recommendations 4 and 5: Strengthening patients and healthcare professional readiness**

Patient readiness should be strengthened through reinforced transnational collaborations of patient organisation, early communication mechanisms, and education programmes enabling patients to recognise emergencies and navigate care during crisis. In parallel, healthcare professional readiness should be enhanced through concise, disease-specific emergency guidelines for rare diseases, available in digital and offline formats such as emergency cards and pocket guides. Targeted training tools should be provided to emergency partners, including emergency physicians, civil protection services, pharmacies, and NGOs, to improve recognition of rare disease vulnerabilities. This implies integration of ERNs into preparedness frameworks. Educational resources should also support HCPs in identifying urgent situations and knowing when to seek expert input. The deployment of mobile diagnostic units, directly connected to ERN specialists, should be explored as a rapid-response solution in crisis settings.

**Recommendation 6: Ensuring digital readiness and resilience**

Digital readiness is essential for crisis response and continuity of care. Efficient digital tools should be designed with built-in redundancy, including national communication systems designed to function independently and avoid cascading failures. Anticipating internet and power outages is critical. Beyond communication, telemedicine should be supported by standardised telepathology and teleradiology networks, enabling real-time expert review of complex cases when on-site expertise is unavailable.

**Recommendation 7: Supporting continuity of international clinical trials**

To mitigate the impact of crisis on rare disease clinical research, ERNs should collaborate with regulators and sponsors to pre-approve adaptive protocols that can be rapidly activated during emergencies. In addition, continuity of international clinical trials can be supported through flexible trial designs, remote monitoring solutions, decentralised assessments, and robust contingency planning.

**Recommendation 8: Establishing a network of molecular diagnostic services**

Cross-border sample transfer, secure data sharing, and harmonised analytical pipelines are essential to maintaining diagnostic capacity during system disruptions. The development of a European network of molecular diagnostic laboratories, able to accept samples from other countries in both short- and long-term crisis scenarios, would ensure cross-border diagnostic capacity during crisis. Participating laboratories would have to comply with recognised quality standards and ensure availability of certified clinical laboratory geneticists and molecular pathologists.

**Recommendation 9: Extending ERN support beyond the European Union**

ERNs emphasised the importance of ensuring that rare disease recommendations and expertise can be mobilised for non-EU countries during crisis. Most ERNs report readiness to support international crisis and disaster responses, as demonstrated during the war in Ukraine, and note that such support, already delivered through patient organisations, collaborative networks, and international clinical trials, could be further strengthened through dedicated mechanisms and resources.

**Recommendation 10: Formally mandate ERNs as crisis response hubs**

ERNs should be formally recognised and resourced as essential crisis response hubs for rare disease populations, supported by dedicated funding. ERNs should be fully integrated into the broader crisis ecosystem, including civil protection authorities, national health ministries, and international organisations involved in emergency response, such as WHO, Médecins Sans Frontières, and the Red Cross. Clear mandates and sustainable resources are necessary to enable ERNs to fulfil this role effectively during future crisis and disasters.

This proposed framework provides a roadmap to strengthen the protection of the estimated 30 million people living with rare diseases in Europe during future crises and disasters. Although developed in the European context, the principles underpinning the plan may also be relevant to rare disease populations globally and, more broadly, to other chronic disease communities.

## Conclusion

ERNs have proven their value as rapid responders during crises affecting rare disease populations. However, transforming reactive capacity into systematic preparedness requires formal mandates, dedicated resources, and integration with national and international emergency frameworks. In this rapidly moving world,^34^^,^^35^ marked by recurrent crises and disasters, we, as ERN coordinators, care for our teams and our patients. Guided by our shared values and our long-term engagement, we remain more than ever committed to safeguarding the rare disease patient community across all contexts.

## Contributors

HD and MS conceived and designed the study. HD, MS, and AA developed the survey instrument. HD coordinated data collection and provided an initial report. HD and MS analysed the data and drafted the manuscript. AA provided critical revision for intellectual content. All 24 ERN coordinators contributed survey responses and reviewed the manuscript. HD and MS directly accessed and verified all data. All authors had full access to all data and had final responsibility for the decision to submit for publication.

## Data sharing statement

Anonymized survey data and the survey instrument are available upon reasonable request to the corresponding author.

## Declaration of interests

**HD** is coordinator of ERN-EYE. **MS** is coordinator of MetabERN. **AA** is coordinator of ERN EpiCARE. All other authors are coordinators of their respective ERNs. **AMP**, **AV**, **CB**, **FH**, **FS**, **GJ**, **IMJM**, **MJLL**, **PF**, **PM**, **RL**, **RMHW**, and **TE** declare no further conflict of interest. **AAMW** reports receiving consulting fees from Rocket Pharma and Solid-BIO, as well as chairing the LEAP trial Data Safety Monitoring Board. **AWL** reports receiving funding from the European Commission as a grant for the coordination of the ERN RARE-LIVER paid to his institution. **HG** reports receiving consulting fees from UCB Pharma, as well as being a member of the N = 1 Collaborative Board of Directors. **JFS** reports receiving funding from the European Commission as a grant for the coordination of the ERN RITA paid to his institution, being a member of the PReS Council, being a member of the data safety monitoring board or advisory board of Bristol Myers Squibb and AlfaSigma paid to his institution, as well as receiving a research grant, consulting fees and honoraria from Pfizer paid to his institution. **JYB** reports receiving funding from the European Commission as a grant for the coordination of the ERN EURACAN paid to his institution, as well as funding from the French National Cancer Institute as a grant for the coordination of the NETSARC+, INTERSARC+ and LYriCAN+ networks paid to his institution. **LS** reports receiving travel and accommodation support from the European Commission for attending the ERN BOND-related meetings and events, as well as being a member of the data safety monitoring board or advisory board of Mereo and Alexion. **MMP** received grants from Agios Pharmaceuticals, Inc., Novartis Pharma AG, Novo Nordisk Health Care A/G, and Pfizer, Inc. with all payments made to their institution. **TOFW** received remuneration from various German courts for expert testimony and from IQWiG for expert assessments related to orphan drug early benefit evaluations under the supervision of the German Federal Ministry of Health, and serves as President of the non-profit organisation FUSE Hessen e.V.
