# Supplementary material for: Crisis readiness for rare disease populations: learnings and recommendations by the European Reference Networks
Source: Lancet Reg Health Eur. 2026 Aug 8;69:101801. doi: 10.1016/j.lanepe.2026.101801 (PMC13476765; doi:10.1016/j.lanepe.2026.101801)
Supplement: Supplementary file 1 [file mmc1.pdf]

Supplementary data

Crisis response and preparedness for Rare Disease populations cross sectional study: Lessons learnt and recommendations by the European Reference Networks

Supplementary table 1. Overview of documents developed by European Reference Networks during the COVID-19 crisis.

ERN	Type	Title	Link
Endo-ERN	List of resources	1. COVID-19 country specific initiatives.<br>2. COVID-19 European initiatives.	1. https://endo-ern.eu/covid-19/country/<br>2. https://endo-ern.eu/covid-19/european-initiatives/
Endo-ERN	Peer-reviewed publications	1. Adrenal function in relation to cytokines and outcome in non-critically ill patients with COVID-19.[1]<br>2. Paradoxical low severity of COVID-19 in Prader-Willi syndrome: data from a French survey on 647 patients.[2]<br>3. Outcome of COVID-19 infections in patients with adrenal insufficiency and excess. Endocrine Connections.[3]<br>4. A critical evaluation of the EU-virtual consultation platform (CPMS) within the European Reference Network on Rare Endocrine Conditions.[4]<br>5. Clinical management of patients with genetic obesity during COVID-19 pandemic: position paper of the ESE Growth & Genetic Obesity COVID-19 Study Group and Rare Endo-ERN main thematic group on Growth and Obesity.[5]	1. https://doi.org/10.1007/s40618-023-02189-y<br>2. https://doi.org/10.1186/s13023-021-01949-4<br>3. https://doi.org/10.1530/EC-22-0416<br>4. https://doi.org/10.1530/EC-22-0281<br>5. https://doi.org/10.1007/s12020-021-02619-y
ERKNet	Peer-reviewed publications	1. COVID-19 in children treated with immunosuppressive medication for kidney diseases.[6]<br>2. The severity of COVID-19 in children on immunosuppressive medication.[7]	1. https://doi.org/10.1136/archdischild-2020-320616<br>2. https://doi.org/10.1016/S2352-4642(20)30145-0
ERN BOND	Peer-reviewed publications	1. Providing high-quality care remotely to patients with rare bone diseases during COVID-19 pandemic.[8]<br>2. The line between COVID-19 pandemic and rare bone diseases.[9]	1. https://doi.org/10.1186/s13023-020-01513-6<br>2. https://doi.org/10.1007/s11845-020-02400-6
ERN EpiCARE	Recommendations	COVID-19 and epilepsy – ERN EpiCARE recommendations.	https://epi-care.eu/covid-19-and-epilepsy-ern-epicare-recommendations/
ERN EpiCARE	List of resources	COVID-19 information for clinicians.	https://www.ilae.org/patient-care/covid-19-and-epilepsy/for-clinicians
ERN eUROGEN	List of resources	COVID-19: eUROGEN statement and resources.	https://eurogen-ern.eu/covid-19/
ERN eUROGEN	Recommendations	EAU COVID-19 rapid reaction recommendations.	https://eurogen-ern.eu/covid19-rapid-reaction-recommendations/
ERN eUROGEN	Webinar	ERN eUROGEN & EAU webinar: COVID-19 & urology.	https://eurogen-ern.eu/ern-eurogen-eau-webinar-covid-19-urology/
ERN EURO-NMD	List of resources	COVID	https://ern-euro-nmd.eu/?s=covid
ERN EURO-NMD	Survey	Results of survey: COVID19 hospital feedback.	https://ern-euro-nmd.eu/news/results-of-survey-covid19-hospital-feedback/?highlight=covid+survey
ERN EURO-NMD	Peer-reviewed publication	Telemedicine in neuromuscular diseases during COVID-19 pandemic: ERN-NMD European survey.[10]	https://doi.org/10.3233/JND-221525
ERN GUARD-Heart	Peer-reviewed publications	1. Hyperinflammatory shock in children during COVID-19 pandemic.[11]<br>2. Kawasaki-like disease: emerging complication during the COVID-19 pandemic.[12]<br>3. SARS-CoV-2, COVID-19, and inherited arrhythmia syndromes.[13]<br>4. COVID-19 vaccination in patients with long QT syndrome.[14]<br>5. European Society of Cardiology guidance for the diagnosis and management of cardiovascular disease during the COVID-19 pandemic: part 1-epidemiology, pathophysiology, and diagnosis.[15]	1. https://doi.org/10.1016/S0140-6736(20)31094-1<br>2. https://doi.org/10.1016/S0140-6736(20)31129-6<br>3. https://doi.org/10.1016/j.hrthm.2020.03.024<br>4. https://doi.org/10.1016/j.hroo.2022.07.011<br>5. https://doi.org/10.1093/eurheartj/ehab697<br>6. https://doi.org/10.1093/eurheartj/ehab696

		6. ESC guidance for the diagnosis and management of cardiovascular disease during the COVID-19 pandemic: part 2-care pathways, treatment, and follow-up.[16]	
ERN PaedCan	Peer-reviewed publications	1. Impact of the COVID pandemic on survivors of childhood cancer and survivorship care: lessons for the future.[17]<br>2. The Global Impact of COVID-19 on Childhood Cancer Outcomes and Care Delivery - A Systematic Review.[18]<br>3. Impacts of COVID-19 on caregivers of childhood cancer survivors.[19]	1. https://doi.org/10.1007/s00520-021-06788-4<br>2. https://doi.org/10.3389/fonc.2022.869752<br>3. https://doi.org/10.1002/pbc.28943
ERN ReCONNET	Poster recommendation	ERN ReCONNET posters and endorsed recommendations on systemic autoimmune diseases and vaccines.	https://reconnet.ern-net.eu/covid-19-ern-reconnet/
ERN ReCONNET	Peer-reviewed publications	1. Long-term outcomes of COVID-19 vaccination in patients with rare and complex connective tissue diseases: The ERN-ReCONNET VACCINATE study.[20]<br>2. ERN ReCONNET points to consider for treating patients living with autoimmune rheumatic diseases with antiviral therapies and anti-SARS-CoV-2 antibody products.[21]<br>3. Shaping the Future of Rare Diseases after a Global Health Emergency: Organisational Points to Consider.[22]	1. https://doi.org/10.1016/j.jtauto.2023.100221<br>2. https://doi.org/10.55563/clinexprheumatol/jpargp<br>3. https://doi.org/10.3390/ijerph17228694
ERN RITA	Webinar	1. Pediatric inflammatory multisystem syndrome (PIMS) in the context of COVID-19.<br>2. COVID-19 vaccination for primary immunodeficiencies & rare autoimmune rheumatic diseases<br>3. Cytokine storm syndromes, including comments on COVID-19/MISC/LongCOVID	1. https://www.youtube.com/watch?v=OYDqY1W2YvU<br>2. https://www.youtube.com/watch?v=tl8ejmUySwo<br>3. https://www.youtube.com/watch?v=yjsKt6hBaLY
ERN RITA	Peer-reviewed publication	The HyperPed-COVID international registry: Impact of age of onset, disease presentation and geographical distribution on the final outcome of MIS-C.[23]	https://doi.org/10.1016/j.jaut.2024.103265
ERN TRANSPLANT-CHILD	Webinar	A webinar on 23 June on "COVID-19 and transplantation in children" in collaboration with ERN Transplant-Child.	https://health.ec.europa.eu/document/download/7d70c844-f923-4986-9fe0-95b2cea348cb_en
ERN TRANSPLANT-CHILD	Filmed interviews	Situation during COVID-19: interviews with our health professionals.	https://transplantchild.eu/situation-during-covid-19-interviews-with-our-health-professionals/
ERN TRANSPLANT-CHILD	Peer-reviewed publication	Pediatric transplantation in Europe during the COVID-19 pandemic: Early impact on activity and healthcare.[24]	https://doi.org/10.1111/ctr.14063
ERN-EuroBloodNet	List of peer-reviewed publications	Disease groups research.	https://eurobloodnet.eu/research/disease-group-research/
ERN-EuroBloodNet	List of resources	COVID-19 and rare haematological diseases.	https://eurobloodnet.eu/covid/covid-19-information/
ERN-EuroBloodNet	Collaborative platform	ERN-EuroBloodNet collaborative platform on red blood cell and COVID-19 patients.	https://eurobloodnet.eu/covid/access-to-the-platform/
ERN-EuroBloodNet	Online training	Provision of Training on Intensive Care Medicine Skills for Health Professionals Not Regularly Working on Intensive Care Units – COVID-19 Skills Preparation Course	https://eurobloodnet.eu/news/160/the-european-commission-together-with-the-european-society-of-intensive-care-medicine-are-supporting-the-eu-healthcare-systems-and-clinicians-dealing-with-the-covid-19-pandemic-on-the-front-line
ERN-EuroBloodNet	Webinar	1. Sickle cell disease patients and parents' patients educational session at ASCAT 2020, a joint project of ERN-EuroBloodNet, ASCAT and BSH<br>2. SCD patients' educational session at ASCAT 2020: webinar on COVID-19 pandemic<br>3. SCD patients' educational session at ASCAT 2020: COVID-19 pandemic patient testimonies from France<br>4. SCD patients' educational session at ASCAT 2020: COVID-19 pandemic patient testimonies from Cyprus	1. https://eurobloodnet.eu/news/168/sickle-cell-disease-patients-and-parents-patients-educational-session-at-ascat-2020-a-joint-project-of-ern-eurobloodnet-ascat-and-bsh<br>2. https://eurobloodnet.eu/education/educational-material/77/scd-patients-educational-session-at-ascat-2020-webinar-on-covid-19-pandemic<br>3. https://eurobloodnet.eu/education/educational-material/66/scd-patients-educational-session-at-ascat-2020-covid-19-pandemic-patient-testimonies-from-france<br>4. https://eurobloodnet.eu/education/educational-material/63/scd-patients-educational-session-at-ascat-2020-covid-19-pandemic-patient-testimonies-from-cyprus

		5. SCD patients' educational session at ASCAT 2020: COVID-19 pandemic patient testimonies from UK<br>6. SCD patients' educational session at ASCAT 2020: COVID-19 pandemic patient testimonies from Portugal<br>7. SCD and COVID-19<br>8. ERN-EuroBloodNet webinars for patients: risk and course of COVID-19 in AA and PNH patients, including vaccination strategies<br>9. Vaccine-induced immune thrombocytopenia and thrombosis (VITT)	5. https://eurobloodnet.eu/education/educational-material/64/scd-patients-educational-session-at-ascat-2020-covid-19-pandemic-patient-testimonies-from-uk<br>6. https://eurobloodnet.eu/education/educational-material/65/scd-patients-educational-session-at-ascat-2020-covid-19-pandemic-patient-testimonies-from-portugal<br>7. https://eurobloodnet.eu/patientsadvocacy/webinar-scd-and-covid-19/<br>8. https://eurobloodnet.eu/education-2/patients/webinars/patients-webinars/risk-and-course-of-covid-19-in-aa-and-pnh-patients/ern-eurobloodnet-webinars-for-patients-risk-and-course-of-covid-19-in-aa-and-pnh-patients-including-vaccination-strategies/<br>9. https://eurobloodnet.eu/education/thursdays-webinars/25/vaccine-induced-immune-thrombocytopenia-and-thrombosis-vitt
ERN-EuroBloodNet	Peer-reviewed publication	COVID- 19 in patients affected by red blood cell disorders, results from the European registry ERN-EuroBloodNet.[25]	https://doi.org/10.1186/s13023-025-03683-7
ERN-EYE (coordinator)	Recommendations	Summary of the view of all ERNs on priorities and contra-indications for COVID-19 vaccinations.	https://endo-ern.eu/wp-content/uploads/2021/02/20210205_summary_ERN_COVID-19_Vaccination_updated20210212.pdf
ERN-LUNG	Online expert advisory platform	Expert Advisory Board EXABO.	https://exabo-lung.mig-frankfurt.de/archive
ERN-RND	Webinar	1. Webinar "COVID-19 and rare diseases"<br>2. Webinar on Huntington's disease & COVID-19	1. https://www.ern-rnd.eu/28-january-2021-webinar-covid-19-and-rare-diseases/<br>2. https://www.ern-rnd.eu/recording-of-webinar-on-huntingtons-disease-covid-19-available/
ERN-Skin	Recommendations	1. Patients with rare skin diseases and COVID-19 virus.<br>2. Vaccination advice.	1. https://ern-skin.eu/covid-19/<br>2. https://ern-skin.eu/vaccination-advices/
VASCERN	List of resources	COVID	https://vascern.eu/?s=covid&post_type=&date

Supplementary table 2. Overview of documents developed by European Reference Networks in response to the war in Ukraine.

ERN	Type	Title	Link
Endo-ERN	Statement	Ukraine and endocrine drug shortages: joint statement from the Ukrainian Diabetology Association, the Association of the Endocrinologists of Ukraine, and the European Society of Endocrinology.	https://www.ese-hormones.org/media/ls0ipjsa/statement-ukraine-drug-shortages-22march2022-v3.pdf
ERKNet	Support	Solidarity with Ukraine #ERNcare4Ua.	https://www.erknet.org/news/solidarity-with-ukraine
ERN eUROGEN	Webinar	Webinar programme.	https://eurogen-ern.eu/webinars/webinar-programme/
ERN EURO-NMD	Congress presentation	Landscape of gene therapy practices across Europe.	https://ern-euro-nmd.eu/wp-content/uploads/2025/03/Landscape-of-gene-therapy-practices-5Mar.pdf
ERN PaedCan	Recommendations	The impact of the war in Ukraine on oncology patients: overview and recommendations for European and Ukrainian health authorities and policymakers.	https://efpia.eu/media/677308/efpia-the-impact-of-the-war-in-ukraine-on-oncology-patients_final.pdf
ERN PaedCan	Support	Ukrainian children living with cancer transferred to Germany from damaged Kyiv paediatric hospital.	https://www.who.int/europe/news/item/19-07-2024-ukrainian-children-living-with-cancer-transferred-to-germany-from-damaged-kyiv-paediatric-hospital
ERN PaedCan	Collaborations	1. Tabletochki: about us.<br>2. St. Jude Children's Research Hospital supports Ukraine cancer patients.<br>3. Childhood Cancer International (CCI) Europe: safe passage out of Ukraine.	1. https://tabletochki.org/en/foundation/<br>2. https://www.stjude.org/about-st-jude/60-minutes-ukraine-segment.html<br>3. https://ccieurope.eu/ukraine/

ERN PaedCan	Peer-reviewed publications	1. Caring for children with cancer evacuated from Ukraine: The patients' perception.[26]<br>2. The impact of armed conflict on global patterns of childhood cancer.[27]<br>3. Psychosocial Impact of the War in Ukraine on Pediatric Cancer Patients and Their Families Receiving Oncological Care Outside Their Country at the Onset of Hostilities.[28]<br>4. Global effort to evacuate Ukrainian children with cancer and blood disorders who have been affected by war.[29]<br>5. Evacuating Patients With Pediatric Cancer From Ukraine: Impact on Medical Care Capacity in Poland.[30]<br>6. Development of a centralised triage centre for children with cancer and blood disorders in response to the humanitarian crisis in Ukraine.[31]	1. https://doi.org/10.1002/pbc.31087<br>2. https://doi.org/10.1016/S1470-2045(24)00559-X<br>3. https://doi.org/10.1016/j.adro.2022.100957<br>4. https://doi.org/10.1016/S2352-3026(22)00259-9<br>5. https://doi.org/10.1200/GO.24.00046<br>6. https://doi.org/10.1016/S1470-2045(23)00456-4
ERN-EuroBloodNet	Support	ERN-EuroBloodNet Patients Assistance Infopoint.	https://eurobloodnet.eu/cross-border-health/ern-eurobloonet-patients-assistance-infopoint/
ERN-EuroBloodNet	Collaboration	Ukraine's patients association for paroxysmal nocturnal hemoglobinuria (PNH).	https://pnh.org.ua/
ERN-EYE (coordinator)	Statement	Statement to support people with rare diseases and complex conditions affected by the war in Ukraine.	https://ern-lung.eu/wp-content/uploads/2022/04/Statement-ERNcare4ua-14-March-2022-2.pdf
ERN-EYE (coordinator)	Website	#ERNcare4Ua Rare Diseases Doctors.	https://www.erncare4ua.com/
ERN-EYE (coordinator)	Peer-reviewed publication	The European Reference Networks for rare and complex diseases respond to the Ukrainian crisis.[32]	https://doi.org/10.1016/j.lanepe.2022.100464
ERN-LUNG	Webinar + on-site training	ERN-LUNG Academy.	https://ern-lung.eu/our-expertise/education/ern-lung-academy/

Supplementary table 3. Overview of documents developed by European Reference Networks in response to other crises.

ERN	Type	Title	Link
ERN Collaboration	Peer-reviewed publication	Mapping the rare disease paediatric clinical trial availabilities in Europe.[33]	https://doi.org/10.3389/fped.2025.1523847
ERN-EuroBloodNet	Congress presentation	SCD recommendations in the EU: a EuroBloodNet review of national implementation, gaps, and research priorities.	https://ascatconferences.com/wp-content/uploads/2025/09/ASCAT-2025-Accepted-Abstract-Submissions.pdf
ERN-EuroBloodNet	Regulatory collaboration	The AEMPS reports the supply of Hydrea 500 mg through the application of Medicines in Special Situations.	https://www.aemps.gob.es/informa/la-aemps-informa-del-suministro-de-hydrea-500-mg-a-traves-de-la-aplicacion-de-medicamentos-en-situaciones-especiales/

Supplementary bibliography

1. Athanasiou N, Diamantopoulos A, Keskinidou C, et al. Adrenal function in relation to cytokines and outcome in non-critically ill patients with COVID-19. J Endocrinol Invest. 2023 Sep 13;47(3):721–8. doi:10.1007/s40618-023-02189-y

2. Coupaye M, Laurier V, Benvegnu G, Poitou C, et al. Paradoxical low severity of COVID-19 in Prader-Willi syndrome: data from a French survey on 647 patients. Orphanet J Rare Dis. 2021 Dec;16(1):325. doi:10.1186/s13023-021-01949-4

3. Nowotny HF, Bryce J, Ali SR, et al. Outcome of COVID-19 infections in patients with adrenal insufficiency and excess. Endocrine Connections. 2023 Jan 30;12(4):e220416. doi:10.1530/EC-22-0416

4. White EK, Wagner IV, Van Beuzekom C, et al. A critical evaluation of the EU-virtual consultation platform (CPMS) within the European Reference Network on Rare Endocrine Conditions. Endocrine Connections. 2022 Nov 1;11(11):e220281. doi:10.1530/EC-22-0281

5. De Groot CJ, Poitou Bernert C, Coupaye M, et al. Clinical management of patients with genetic obesity during COVID-19 pandemic: position paper of the ESE Growth & Genetic Obesity COVID-19 Study Group and Rare Endo-ERN main thematic group on Growth and Obesity. Endocrine. 2021 Mar;71(3):653–62. doi:10.1007/s12020-021-02619-y PubMed PMID: 33512658; PubMed Central PMCID: PMC7845285.

6. Marlais M, Wlodkowski T, Al-Akash S, et al. COVID-19 in children treated with immunosuppressive medication for kidney diseases. Arch Dis Child. 2021 Jul 19;106(8):798–801. doi:10.1136/archdischild-2020-320616 PubMed PMID: 33355203; PubMed Central PMCID: PMC7754669.

7. Marlais M, Wlodkowski T, Vivarelli M, et al. The severity of COVID-19 in children on immunosuppressive medication. The Lancet Child & Adolescent Health. 2020 Jul;4(7):e17–8. doi:10.1016/S2352-4642(20)30145-0

8. Brizola E, Adami G, Baroncelli GI, et al. Providing high-quality care remotely to patients with rare bone diseases during COVID-19 pandemic. Orphanet J Rare Dis. 2020 Dec;15(1):228. doi:10.1186/s13023-020-01513-6

9. Sangiorgi L, Brizola E, COVID-19 Helpline for Rare Bone Diseases Group. The line between COVID-19 pandemic and rare bone diseases. Ir J Med Sci. 2021 Aug;190(3):1243–4. doi:10.1007/s11845-020-02400-6 PubMed PMID: 33140293; PubMed Central PMCID: PMC7605471.

10. El-Hassar L, Amara A, Sanson B, et al. Telemedicine in Neuromuscular Diseases During Covid-19 Pandemic: ERN-NMD European Survey. J Neuromuscul Dis. 2023;10(2):173–84. doi:10.3233/JND-221525 PubMed PMID: 36373291.

11. Riphagen S, Gomez X, Gonzalez-Martinez C, Wilkinson N, Theocharis P. Hyperinflammatory shock in children during COVID-19 pandemic. The Lancet. 2020 May;395(10237):1607–8. doi:10.1016/S0140-6736(20)31094-1

12. Viner RM, Whittaker E. Kawasaki-like disease: emerging complication during the COVID-19 pandemic. The Lancet. 2020 Jun;395(10239):1741–3. doi:10.1016/S0140-6736(20)31129-6

13. Wu CI, Postema PG, Arbelo E, et al. SARS-CoV-2, COVID-19, and inherited arrhythmia syndromes. Heart Rhythm. 2020 Sep;17(9):1456–62. doi:10.1016/j.hrthm.2020.03.024 PubMed PMID: 32244059; PubMed Central PMCID: PMC7156157.

14. Wu CI, Schwartz PJ, Ackerman MJ, Wilde AAM. COVID-19 vaccination in patients with long QT syndrome. Heart Rhythm O2. 2022 Dec;3(6):706–9. doi:10.1016/j.hroo.2022.07.011

15. The Task Force for the management of COVID-19 of the European Society of Cardiology, Baigent C, Windecker S, et al. European Society of Cardiology guidance for the diagnosis and management of cardiovascular disease during the COVID-19 pandemic: part 1—epidemiology, pathophysiology, and diagnosis. European Heart Journal. 2022 Mar 14;43(11):1033–58. doi:10.1093/eurheartj/ehab696

16. The Task Force for the management of COVID-19 of the European Society of Cardiology, Baigent C, Windecker S, et al. ESC guidance for the diagnosis and management of cardiovascular disease during the COVID-19 pandemic: part 2—care pathways, treatment, and follow-up. European Heart Journal. 2022 Mar 14;43(11):1059–103. doi:10.1093/eurheartj/ehab697

17. Prasad M, Goswami S, Deodhar J, Chinnaswamy G. Impact of the COVID pandemic on survivors of childhood cancer and survivorship care: lessons for the future. Support Care Cancer. 2022 Apr;30(4):3303–11. doi:10.1007/s00520-021-06788-4 PubMed PMID: 34985560; PubMed Central PMCID: PMC8727237.

18. Majeed A, Wright T, Guo B, Arora RS, Lam CG, Martiniuk AL. The Global Impact of COVID-19 on Childhood Cancer Outcomes and Care Delivery - A Systematic Review. Front Oncol. 2022 Apr 7;12:869752. doi:10.3389/fonc.2022.869752

19. Wimberly CE, Towry L, Caudill C, Johnston EE, Walsh KM. Impacts of COVID-19 on caregivers of childhood cancer survivors. Pediatric Blood & Cancer. 2021 Apr;68(4):e28943. doi:10.1002/pbc.28943

20. Tani C, Cardelli C, Depascale R, et al. Long-term outcomes of COVID-19 vaccination in patients with rare and complex connective tissue diseases: The ERN-ReCONNET VACCINATE study. J Transl Autoimmun. 2023 Dec;7:100221. doi:10.1016/j.jtauto.2023.100221 PubMed PMID: 38162456; PubMed Central PMCID: PMC10755036.

21. Talarico R, Ramirez GA, Barreira SC, et al. ERN ReCONNET points to consider for treating patients living with autoimmune rheumatic diseases with antiviral therapies and anti-SARS-CoV-2 antibody products. Clinical and Experimental Rheumatology. 2023 Mar 2. doi:10.55563/clinexprheumatol/jpargp

22. Talarico R, Marinello D, Cannizzo S, et al. Shaping the Future of Rare Diseases after a Global Health Emergency: Organisational Points to Consider. IJERPH. 2020 Nov 23;17(22):8694. doi:10.3390/ijerph17228694

23. Caorsi R, Consolaro A, Speziani C, et al. The HyperPed-COVID international registry: Impact of age of onset, disease presentation and geographical distribution on the final outcome of MIS-C. Journal of Autoimmunity. 2024 Jul;147:103265. doi:10.1016/j.jaut.2024.103265

24. Doná D, Torres Canizales J, Benetti E, et al. Pediatric transplantation in Europe during the COVID-19 pandemic: Early impact on activity and healthcare. Clinical Transplantation. 2020 Oct;34(10):e14063. doi:10.1111/ctr.14063

25. Velasco Puyo P, Christou S, Campisi S, et al. COVID- 19 in patients affected by red blood cell disorders, results from the European registry ERN-EuroBloodNet. Orphanet J Rare Dis. 2025 Apr 16;20(1):183. doi:10.1186/s13023-025-03683-7

26. Bolognese M, Massimino M, Cabibbe D, et al. Caring for children with cancer evacuated from Ukraine: The patients' perception. Pediatric Blood & Cancer. 2024 Aug;71(8):e31087. doi:10.1002/pbc.31087

27. Espinoza P, Rice HE, Wise PH, et al. The impact of armed conflict on global patterns of childhood cancer. The Lancet Oncology. 2025 Apr;26(4):421–4. doi:10.1016/S1470-2045(24)00559-X

28. Uğurluer G, Özyar E, Corapcioglu F, Miller RC. Psychosocial Impact of the War in Ukraine on Pediatric Cancer Patients and Their Families Receiving Oncological Care Outside Their Country at the Onset of Hostilities. Adv Radiat Oncol. 2022;7(4):100957. doi:10.1016/j.adro.2022.100957 PubMed PMID: 35865369; PubMed Central PMCID: PMC9294973.

29. Agulnik A, Kizyma R, Salek M, et al. Global effort to evacuate Ukrainian children with cancer and blood disorders who have been affected by war. The Lancet Haematology. 2022 Sep;9(9):e645–7. doi:10.1016/S2352-3026(22)00259-9

30. Oszer A, Kliuchkivska K, Kołodrubiec J, et al. Evacuating Patients With Pediatric Cancer From Ukraine: Impact on Medical Care Capacity in Poland. JCO Glob Oncol. 2025 Nov;(11):e2400046. doi:10.1200/GO.24.00046

31. Salek M, Mueller A, Alanbousi I, et al. Development of a centralised triage centre for children with cancer and blood disorders in response to the humanitarian crisis in Ukraine. The Lancet Oncology. 2023 Dec;24(12):1315–8. doi:10.1016/S1470-2045(23)00456-4

32. Dollfus H, Arzimanoglou A, Scarpa M. The European Reference Networks for rare and complex diseases respond to the Ukrainian crisis. The Lancet Regional Health - Europe. 2022 Aug;19:100464. doi:10.1016/j.lanepe.2022.100464

33. Degraeuwe E, Turner MA, Fernandes RM, Raes A, Vande Walle J, Schaefer F. Mapping the rare disease paediatric clinical trial availabilities in Europe. Front Pediatr. 2025 May 19;13:1523847. doi:10.3389/fped.2025.1523847

Supplementary material 1. Cross-sectional online survey sent to ERN coordinators to assess their experiences, responses, challenges, and recommendations for crisis and disaster preparedness.

ERN Survey on Rare Diseases and Crisis /Disaster

Fields marked with * are mandatory.

ERN Survey on Rare Diseases and Crisis /Disaster

This survey is aimed at **ERN coordinators** (or delegated to an ERN senior MD) and in close collaboration with the Project Managers, to assess past experiences and anticipated needs of ERNs in the context of Crisis /Disaster situations occurring in an EU country or border countries.

One reply is expected per ERN. Your participation is highly awaited and all ERNs will be of course cited in the context of this collective work.

The survey also takes place in the context of JARDIN WP6.6 (Activity leads: Pr H Dollfus, ERN-EYE and Pr M Scarpa, MetabERN).

The ultimate goal will be to draw a preparedness plan dedicated to rare diseases specificities in the context of crisis/disaster based on the ERN's expertise and the available tools (CPMS2.0, recommendations/guidelines, etc.). Five main axes are anticipated:

1) Ensure continuity of care (Care Continuity Plans)
2) Patient preparedness
3) Facing Drug shortages
4) Rare diseases awareness / training / education of all emergency partners
5) Facing Trials interruptions.

Each question follows a Yes/No format or ranking of items but also includes space for comments and references. **Do not hesitate to fill the free text boxes and add references**. Although some questions may seem trivial, they are important for statistical purposes. Please ensure that all questions are answered.

Many thanks for your contribution!

GENERAL INFORMATION

About you (ERN coordinator or representative)

* First name:

* Last name:

* Email address:

* Country:
- Afghanistan
- Albania
- Algeria
- Andorra
- Angola
- Antigua and Barbuda
- Argentina
- Armenia
- Australia
- Austria
- Azerbaijan

- Bahamas
- Bahrain
- Bangladesh
- Barbados
- Belarus
- Belgium
- Belize
- Benin
- Bhutan
- Bolivia
- Bosnia and Herzegovina
- Botswana
- Brazil
- Brunei Darussalam
- Bulgaria
- Burkina Faso
- Burundi
- Cabo Verde
- Cambodia
- Cameroon
- Canada
- Central African Republic
- Chad
- Chile
- China
- Colombia
- Comoros
- Congo
- Costa Rica
- Côte D'Ivoire
- Croatia
- Cuba
- Cyprus
- Czechia
- Democratic Republic of the Congo
- Denmark
- Djibouti
- Dominica
- Dominican Republic
- Ecuador
- Egypt
- El Salvador

Equatorial Guinea
- Eritrea
- Estonia
- Eswatini
- Ethiopia
- Fiji
- Finland
- France
- Gabon
- Gambia
- Georgia
- Germany
- Ghana
- Greece
- Grenada
- Guatemala
- Guinea
- Guinea Bissau
- Guyana
- Haiti
- Honduras
- Hungary
- Iceland
- India
- Indonesia
- Iran
- Iraq
- Ireland
- Israel
- Italy
- Jamaica
- Japan
- Jordan
- Kazakhstan
- Kenya
- Kiribati
- Kuwait
- Kyrgyzstan
- Laos
- Latvia
- Lebanon
- Lesotho

Liberia
- Libya
- Liechtenstein
- Lithuania
- Luxembourg
- Madagascar
- Malawi
- Malaysia
- Maldives
- Mali
- Malta
- Marshall Islands
- Mauritania
- Mauritius
- Mexico
- Micronesia
- Monaco
- Mongolia
- Montenegro
- Morocco
- Mozambique
- Myanmar
- Namibia
- Nauru
- Nepal
- Netherlands
- New Zealand
- Nicaragua
- Niger
- Nigeria
- North Korea
- North Macedonia
- Norway
- Oman
- Pakistan
- Palau
- Panama
- Papua New Guinea
- Paraguay
- Peru
- Philippines
- Poland

- Portugal
- Qatar
- Republic of Moldova
- Romania
- Russian Federation
- Rwanda
- Saint Kitts and Nevis
- Saint Lucia
- Saint Vincent and the Grenadines
- Samoa
- San Marino
- Sao Tome and Principe
- Saudi Arabia
- Senegal
- Serbia
- Seychelles
- Sierra Leone
- Singapore
- Slovakia
- Slovenia
- Solomon Islands
- Somalia
- South Africa
- South Korea
- South Sudan
- Spain
- Sri Lanka
- Sudan
- Suriname
- Sweden
- Switzerland
- Syrian Arab Republic
- Tajikistan
- Tanzania
- Thailand
- Timor-Leste
- Togo
- Tonga
- Trinidad and Tobago
- Tunisia
- Türkiye
- Turkmenistan

Tuvalu
- Uganda
- Ukraine
- United Arab Emirates
- United Kingdom
- United States of America
- Uruguay
- Uzbekistan
- Vanuatu
- Venezuela
- Viet Nam
- Yemen
- Zambia
- Zimbabwe

* Name of your European Reference Network:

* I agree to be contacted again if further information or clarification is needed following this survey.

Yes No

About your ERN

* Has your ERN ever experienced a crisis or disaster situation (**excluding the COVID-19 pandemic and the war in Ukraine**):

Yes No

Additional comments:

References & Links:

* Can you anticipate major threats that, if your ERN was exposed to, **would lead to challenging situations** (besides COVID pandemic and the War in Ukraine)?

- Natural disasters (e.g. earthquakes, floods, wildfires)
- Climate change, particularly extreme heat
- Climate change, particularly extreme cold

- [ ] Infectious disease outbreaks (e.g., COVID-19 or similar pandemics)
- [ ] Damage to infrastructure (e.g., roads, hospitals), preventing physical access to car
- [ ] Cyberattacks affecting hospital systems or ERN infrastructure
- [ ] Breakdown of digital tools essential to ERN operations (e.g. CPMS, data platforms)
- [ ] Major energy or utility shortages (e.g. electricity, water, heating)
- [ ] Political instability or changes in healthcare policy
- [ ] Major healthcare workforce shortages
- [ ] Disruption of medical supply chains (e.g. equipment, drugs)
- [ ] Cross-border mobility restrictions (e.g. due to new border regulations)
- [ ] Other (please specify)

Additional comments:

References & Links:

Can you rank the top five diseases (or groups of diseases) within your ERN that would require urgent attention during a crisis or disaster (e.g., due to risk of rapid deterioration or death without timely diagnosis or care)?

	Disease / Group of diseases
1 *	
2 *	
3 *	
4 *	
5 *	

Additional comments:

* What would be the biggest consequences for your ERN diseases in case of crisis/disaster? *Please rank 1-6, 1 being the highest*:

Use drag&drop or the up/down buttons to change the order or accept the initial order.

- Interruption of the **medical care pathway** (e.g., no access to outpatient services or hospital care at ERN expert centres)
- Interruption of **surgical care pathway** (e.g., no access to planned or emergency surgeries at ERN expert centres)
- Lack of **access to essential medical devices** (e.g., ventilators, dialysis equipment, etc.)
- **Shortages or discontinuation of essential medicines** (e.g., for oncology, epilepsy, etc.)
- **Disruption of clinical trials** or access to innovative therapies
- **Shortage of healthcare professionals** (e.g., doctors, nurses, rehabilitation staff, etc.)

Additional comments:

* Are there crisis or disaster-related risks linked to **climate change** that are particularly relevant to the diseases covered by your ERN (e.g., heat sensitivity in skin or neurological conditions)?

Yes No Not applicable

Additional comments:

References & Links:

* In the context of your ERN's disease area, could a crisis or disaster create the **need for urgent or semi-urgent cross-border physical transfer of patients** to one of your ERN HCPs (e.g., for urgent transplantation) due to a disruption of care in another EU country?

Yes No Not applicable

Additional comments:

References & Links:

ERN RESPONSE TO THE COVID-19 PANDEMIC

Impact of the COVID-19 pandemic

* Did the COVID-19 pandemic globally have an impact on your ERN?

Yes No Not applicable

Additional comments:

References & Links:

* Did the COVID-19 pandemic generate additional activities at the level of **your ERN management team?**

Yes No Not applicable

Additional comments:

References & Links:

* Did the COVID-19 pandemic **affect the care pathways** of your ERN as a whole?

Yes No Not applicable

Additional comments:

References & Links:

* Did you observe that the HCP members of your ERN were able to adapt quickly to the pandemic situation?

Yes No Not applicable

Additional comments:

References & Links:

* Did you observe differences between countries in how HCP members adapted to the pandemic situation?

Yes No Not applicable

Additional comments:

References & Links:

* During the COVID-19 pandemic, what were the most significant impacts on your ERN activities? *Please rank the following from 1 to 6 (1 = most significant)*:

Use drag&drop or the up/down buttons to change the order or accept the initial order.

- Interruption of care continuity (e.g., no access to HCPs, missed diagnostic or follow-up visits, cancelled hospitalizations)
- Lack of patient education, information, or communication
- Drug shortages
- Urgent need to train emergency teams or non-specialist physicians
- Interruption of clinical trials

Other (please specify)

Please specify "other":

Additional comments:

References & Links:

Measures taken by your ERN during the COVID-19 Pandemic

What actions did your ERN take in response to COVID-19 during the pandemic? Please answer the following items and provide any supporting comments or references.

* Did your ERN produce and share specific communication materials **for patients**? *If possible, please include links or attach relevant documents.*

Yes No Not applicable

Additional comments:

References & Links:

* Did your ERN **work directly with patient groups** during the pandemic? *If yes, please provide details in the comments.*

Yes No Not applicable

Additional comments:

References & Links:

* Did your ERN produce and share specific communication materials **for healthcare professionals**? *If possible, please include links or attach relevant documents.*

Yes No Not applicable

Additional comments:

References & Links:

* Did your ERN increase its use of the **CPMS platform** as a communication tool for patient care during the pandemic?

Yes No Not applicable

Additional comments:

References & Links:

* Did your ERN establish contact with any **official stakeholders** dedicated to the COVID-19 emergency response, on addition to the DG Santé (e.g., Health Ministry, DG ECHO, Médecins Sans Frontières, Red Cross, NGOs, etc.) to improve care pathways in your field?

Yes No Not applicable

Additional comments (please specify stakeholders):

References & Links:

ERN RESPONSE TO THE WAR IN UKRAINE

Acute Phase: February – June 2022

Please answer the following questions regarding the initial response of your ERN and its HCP members to the outbreak of the war in Ukraine. When relevant, include comments, examples, or links to supporting materials.

* Did the start of the war in Ukraine generate additional activities at the level of your **ERN management team**?

○ Yes ○ No ○ Not applicable

Additional comments:

References & Links:

What types of activities were undertaken i**n addition to the usual ones** by your ERN during this period?

* Increased communication and information efforts

○ Yes ○ No ○ Not applicable

Additional comments:

References & Links:

* Guidance for establishing contacts (e.g., for patient transfers from Ukraine to the EU)

○ Yes ○ No ○ Not applicable

Additional comments:

References & Links:

* Provision of remote medical advice (via CPMS or other channels)

Yes No Not applicable

Additional comments:

References & Links:

* Establishing or receiving contact with Ukrainian patient associations

Yes No Not applicable

Additional comments:

References & Links:

* Being informed of drug shortages in Ukraine and helping identify solutions?

Yes No Not applicable

Additional comments:

References & Links:

* Did your ERN have **direct contact points with stakeholders in Ukraine** (for transfers, clinical advice, etc.)?

Yes No Not applicable

Additional comments:

References & Links:

* Did your ERN have **contact points** in **border countries** (EU or non-EU) to support transfer or evacuation?

Yes No Not applicable

Additional comments:

References & Links:

* Did the beginning of the war generate additional activities at the level of your ERN's **HCP members**?

Yes No Not applicable

Additional comments:

References & Links:

* Were your HCP centres involved in **providing medical care to displaced persons** from Ukraine in their country?

Yes No Not applicable

Additional comments:

References & Links:

* Did your centres provide **online medical opinions** for Ukrainian patients (e.g., via CPMS or other platforms)?

Yes No Not applicable

Additional comments:

References & Links:

---

* **What specific measures** did your ERN take during the **first three months of the war**?

- Development of ERN-specific communication materials (e.g., website updates, translations)
- Use of CPMS for case discussions involving Ukrainian patients?
- Participation in the joint ERN response with website initiatives
- Collaboration with external organizations (e.g., patient groups, learned societies, ministries, MSF, Red Cross, DG ECHO, DG SANTE, etc.)

Additional comments:

References & Links:

Ongoing War in Ukraine – Chronic Phase (since Summer 2022, including the Kyiv Hub Creation)

Please respond to the following questions regarding the ongoing impact of the war in Ukraine on your ERN, particularly since summer 2022 and the establishment of the Rare Disease Hub at Ohmatdyt Children's Hospital in Kyiv. When applicable, provide comments or links to supporting materials.

* Do you currently have contacts with **the Ukrainian Hub** (Ohmatdyt Children's Hospital, Kyiv)?

Yes No

References & Links:

---

* Does your ERN have any other care **contact points in Ukraine besides the Kyiv Hub**?

Yes No

Additional comments:

References & Links:

---

* **As of today**, has the creation of the **Rare Disease Hub in Kyiv** changed your ERN's activities for Ukrainian patients, particularly in terms of referrals or CPMS 2.0 case discussions?

Yes No

Additional comments:

References & Links:

---

* **As of today**, are referrals or medical opinions for Ukrainian patients currently being managed **through other channels** (e.g., learned societies, informal expert networks)?

Yes No

Additional comments:

References & Links:

---

* Does the ongoing war in Ukraine **currently affect** your **ERN management team**'s activities (e.g., time spent, translations, coordination, exchanges, etc.)?

Yes No

Additional comments:

References & Links:

* **As of today**, does the war in Ukraine continue to impact the activities of your ERN's **HCPs**?

- Yes - in a major way
- Yes - in a moderate way
- Yes - in a minimal way
- Impact varies depending on the country of the HCP
- No

* **Currently**, are you informed of any specific needs related to Ukrainian patients in your ERN's field (e.g., drug shortages)?

Yes No

Additional comments:

References & Links:

* **Currently**, is your ERN collaborating with any other organizations due to the war in Ukraine (e.g., patient groups, learned societies, MSF, Red Cross, WHO, DG ECHO, DG SANTE, national health ministries)?

Yes No

Additional comments:

References & Links:

Long-Term Engagement and Support for Ukraine (Since 2022)

* Since 2022, have any Ukrainian healthcare professionals been hosted by HCPs within your ERN?

Yes No

Additional comments:

References & Links:

* Has your ERN translated any materials into Ukrainian (including website content)?

Yes No Not applicable

Additional comments:

References & Links:

* Has your ERN provided training materials or free access to meetings for Ukrainian colleagues or patient representatives?

Yes No Not applicable

Additional comments:

References & Links:

* In your opinion, what have overall been the greatest challenges in your ERN's field regarding **Ukraine since 2022**? *Please rank the following from 1 to 6 (1 = most significant challenge)*:

Use drag&drop or the up/down buttons to change the order or accept the initial order.

- Disruption of care continuity
- Drug shortages
- Training needs for Ukrainian professionals

- Interruption of clinical trials
- Ongoing need for patient transfers outside Ukraine
- Demand for CPMS use

UNDERSTANDING ERN NEEDS AND PREPAREDNESS

This section focuses on determining your ERN needs during a crisis, how your ERN has experienced or prepared for crisis, and the strategies used to address those needs. Please respond based on both past experiences and current plans.

Care Continuity

Did your ERN have pre-established **care continuity plans** for crises (for example, through learned societies or other relevant institutions) before experiencing them?

- Yes, comprehensive plans were in place
- No, plans were developed during the crisis
- Yes, but only partial/limited plans
- No plans were developed

How would you rate your ERN **care continuity plans**' overall effectiveness during crisis periods:

- Not effective
- Slightly effective
- Moderately effective
- Very effective
- Extremely effective
- Room for improvement

Does your ERN currently have a plan in place to support **care continuity** in crisis or disaster situations (after having experienced COVID-19 and Ukraine)?

- Yes, a comprehensive plan is in place
- No, but a plan is under development
- Yes, but only partial or limited measures
- No, no plan currently exists

* What were the main challenges, or what do you expect the main challenges to be, in maintaining **care continuity**? *(Select the top 3 options)*

Maximum 3 selection(s)

- Physical access to healthcare facilities
- Staff availability
- Communication barriers

- Medication/treatment access
- Diagnostic services disruption
- Cross-border care coordination
- Data sharing limitations
- Funding/resource constraints
- Other

---

* What elements has your ERN included, or is planning to include, in your **care continuity plans**? (Select all options that apply)

- Virtual consultation capabilities
- Cross-border patient transfer protocols
- Medication access contingencies
- Alternative treatment facilities identification
- Patient communication systems
- Data access/sharing provisions
- Staff redeployment strategies
- Other

---

* In the context of a crisis or disaster, could genetic testing play a role in your ERN's field, and would access to surrogate laboratories be useful in such situations?

Yes No Not applicable

Additional comments:

References & Links:

Patient Preparedness

Did your ERN have **pre-established patient preparedness plans** for crises before experiencing them?

- Yes, comprehensive plans were in place
- No, plans were developed during the crisis
- Yes, but only partial/limited plans
- No plans were developed

Based on past experiences, which **patient preparedness** measures have been most effective during l crises /disaster situations? *(Select the top 3 options)*

Maximum 3 selection(s)

- [ ] Emergency medication supplies
- [ ] Easy access to medical documentation
- [ ] Remote patient monitoring capabilities
- [ ] Reliable patient–provider communication channels
- [ ] Patient self-care education
- [ ] Peer support or patient networks
- [ ] Connection to local emergency services
- [ ] Other

Does your ERN currently have a plan in place to support **patient preparedness** in crisis or disaster situations?

- ○ Yes, a comprehensive plan is in place
- ○ No, but a plan is under development
- ○ Yes, but only partial or limited measures
- ○ No, no plan currently exists

---

* What types of support has your ERN used or is planning to use **to help patients** to prepare for crises? *(Select all options that apply)*

- [ ] Emergency preparedness education materials
- [ ] Individual emergency care plans
- [ ] Guidance on medication stockpiling
- [ ] Training on treatment self-administration
- [ ] Systems for storing and sharing emergency contact information
- [ ] Patient-held medical records or care passports
- [ ] Virtual support groups or peer-to-peer resources
- [ ] Other

How would you rate your ERN's overall effectiveness in supporting **patient preparedness** during crises?

- ○ Not effective
- ○ Slightly effective
- ○ Moderately effective
- ○ Very effective
- ○ Extremely effective
- ○ Room for improvement

---

* Has your ERN involved **patients or patient organizations** in designing or improving preparedness strategies?

○ Yes ○ No ○ Not applicable

Additional comments:

References & Links:

Preparedness and Response to Drug Shortages

In the context of your ERN's disease area, are there any essential components of care **whose unavailability would pose life-threatening risks or cause severe complications**, requiring urgent response measures for your patient population? *(Please select as many options as applicable)*

- ☐ **Essential drugs unavailability would pose life-threatening risks or cause severe complications**
- ☐ **Specific treatments** (e.g transfusions) **unavailability would pose life-threatening risks or cause severe complications**
- ☐ **Specific diets unavailability would pose life-threatening risks or cause severe complications**
- ☐ **Specialized medical devices** (e.g. dialysis) **unavailability would pose life-threatening risks or cause severe complications, innovative therapies**
- ☐ No/ Not applicable

Additional comments:

References & Links:

Has your ERN experienced rare disease medication shortages during crises (e.g. Covid-19, war in Ukraine)?

- ○ No shortages experienced
- ○ Minor shortages with minimal impact
- ○ Moderate shortages with some patient impact
- ○ Severe shortages with significant patient impact
- ○ Critical shortages threatening patient lives

How effective was your ERN's response to medication shortages during crisis?

- ○ Not effective
- ○ Slightly effective
- ○ Moderately effective

- Very effective
- Extremely effective
- Room for improvement

---

* What strategies has your ERN used or is planning to use to address **medication shortages**, whether in past or potential future crises? *(Select all options that apply)*

- Cross-border medication sourcing
- Alternative medication protocols
- Compounding pharmacies engagement
- Prioritization systems for limited supplies
- Extended prescription durations
- Coordinated advocacy to manufacturers/authorities
- Patient-level medication sharing systems
- Other

Rare Disease Awareness among Emergency Partners

* Which emergency partners received or are planned to receive rare disease training or awareness materials from your ERN? *(Select all options that apply)*

- Emergency medical services
- Emergency department staff
- Disaster response teams
- Humanitarian aid organizations
- Crisis shelters/evacuation centres
- Military medical personnel
- Civil defence/protection agencies
- Governmental emergency management
- Other
- No partners have been identified yet

Additional comments:

---

* Did your ERN provide or is planning to provide **education or training on rare diseases** to emergency partners prior to crises?

- No training provided
- Limited training to select partners provided/planned
- Moderate training to multiple partners provided/planned

- Comprehensive training programs provided/planned

---

* What educational methods did your ERN use or is planning to use **for emergency partners?** *(Select all options that apply)*

- Emergency care cards/protocols
- Online training modules
- In-person workshops
- Consultation hotlines
- Quick reference guides
- Mobile applications
- Integration in emergency medicine curricula
- Other
- No methods have been identified yet

Additional comments:

How would you rate **the preparedness of emergency partner**s to address rare disease needs during crises?

- Not prepared
- Slightly prepared
- Moderately prepared
- Very prepared
- Extremely prepared

What could be the most **critical knowledge gaps among emergency partners** regarding rare diseases?

What improvements would you suggest for future rare disease **education of emergency partners**?

Clinical Trials Continuity

* In the event of a crisis or disaster, could the interruption of clinical trials pose life-threatening risks or lead to severe complications for patients within your ERN, requiring urgent intervention?

- Yes
- No
- Not applicable

Additional comments:

References & Links:

Were clinical trials for rare diseases within your ERN interrupted during crises?

- No trials were active during crises (COVID-19 pandemic, war in Ukraine)
- No interruptions to active trials
- Minor interruptions with quick resumption
- Significant interruptions with eventual resumption
- Permanent termination of some trials
- Unknown

Additional comments:

What percentage of active trials experienced disruptions (COVID-19 pandemic, war in Ukraine)?

- 0%
- 1-25%
- 26-50%
- 51-75%
- >75%
- Unknown

Additional comments:

What were the main challenges to clinical trial continuity (COVID-19 pandemic, war in Ukraine)? *(Select top 3)*

Maximum 3 selection(s)

- [ ] Patient inability to attend in-person visits
- [ ] Investigational product supply chain disruptions

- [ ] Staff redeployment to crisis response
- [ ] Laboratory/diagnostic service limitations
- [ ] Regulatory/ethics committee delays
- [ ] Funding/sponsor issues
- [ ] Data collection/monitoring challenges
- [ ] Site closures/access restrictions
- [ ] Other

Rate the effectiveness of measures taken to ensure clinical trial continuity during previous crisis (for example, COVID-19 pandemic, war in Ukraine):

- ○ Not effective
- ○ Slightly effective
- ○ Moderately effective
- ○ Very effective
- ○ Extremely effective
- ○ Room for improvement

---

* What adaptations did your ERN implement or is planning to implement to **maintain trial continuity**? *(Select all options that apply)*

- [ ] Remote monitoring/telehealth visits
- [ ] Home delivery of investigational products
- [ ] Protocol amendments for flexibility
- [ ] Alternative assessment methods
- [ ] Extended visit windows
- [ ] Decentralized trial approaches
- [ ] Transfer of patients to alternative sites
- [ ] Other

---

* Does your ERN have an overall view or mapping of where rare disease trials are conducted in the EU, including the HCPs involved and their contact details?

○ Yes ○ No ○ Not applicable

Additional comments:

References & Links:

* In the event of clinical trial disruptions, is your ERN likely to be contacted by key stakeholders (e.g., industry, HCPs, patient groups), indicating that it is recognised as a central communication hub in your field?

- Yes
- No
- Not applicable

Additional comments:

References & Links:

What recommendations would you make for clinical trial resilience during future crises?

COMMUNICATION AND DIGITAL TOOLS

Communication

* Which digital tools proved most valuable for your ERN during crises (COVID-A9, war in Ukraine)? *(Select the top 3 options)*

Maximum 3 selection(s)

- Telehealth platforms
- Electronic health records
- Patient registries
- Mobile health applications
- Clinical decision support systems
- Virtual multidisciplinary team meetings
- Social media/messaging platforms
- Other

* Has your ERN **developed any guidelines or recommendations** for **healthcare professionals** on how to manage specific diseases or disease groups during crisis or disaster situations (e.g., power outages, extreme heat)?

- Yes
- No
- Not applicable

Additional comments:

References & Links:

* Has your ERN **developed any guidelines or recommendations** for **patients** on how to manage specific diseases or disease groups during crisis or disaster situations (e.g., power outages, extreme heat)?

Yes No Not applicable

Additional comments:

References & Links:

CPMS

* In your ERN's field, is the current use of CPMS 2.0 suitable for addressing urgent situations (e.g., responses needed within 48 hours)? In other words, if a crisis or disaster occurs, do you think that CPMS 2.0 can be used promptly to support professionals from other HCPs, whether ERN members or not?

Yes No Not applicable

Additional comments:

References & Links:

* In your ERN's field, could CPMS be used to address urgent situations in the future, provided that reactive panels are established? In other words, would your ERN be willing to organise rapid-response panels to handle CPMS cases within a short timeframe during a crisis or disaster?

Yes No Not applicable

Additional comments:

References & Links:

Telecommunications

* Are you aware of which HCPs within your ERN currently have access to a **teleconsultation system** provided by their institution as part of routine care?

Yes No Not applicable

Additional comments:

References & Links:

* In the event of a crisis or disaster where patients are unable to travel, would HCPs within your ERN need to connect directly with patients via **telemedicine**?

Yes No Not applicable

Additional comments:

References & Links:

* In your ERN’s field, are there any tools—besides CPMS 2.0—that can be used by exposed medical doctors and healthcare professionals to seek expert opinions, **assuming internet access is available**?

Yes No Not applicable

Additional comments:

References & Links:

* In your ERN’s field, are you aware of the needs and available tools for medical doctors and healthcare professionals seeking expert opinions during a crisis or disaster **when internet access is NOT available**?

Yes No Not applicable

Additional comments:

References & Links:

* In the context of a crisis or disaster where patients are unable to travel, would there be a need to **provide a telemedicine system** to your ERN or to some of its member HCPs?

Yes No Not applicable

Additional comments:

References & Links:

CONCLUSION AND FURTHER DEVELOPMENTS

Based on your experience, what are your top three recommendations for improving ERN response to future crises?

	.
1	
2	
3	

* This survey has focused on patients living in the EU affected by crisis or disaster situations. Would your ERN be prepared to provide support to patients located **outside the EU** in such contexts?

Yes No Not applicable

Additional comments:

References & Links:

* Does your ERN have any ongoing or established contacts with international organizations involved in crisis or disaster response (e.g., Médecins Sans Frontières, WHO, Red Cross, etc.)?

Yes No Not applicable

Additional comments:

References & Links:

* Is your ERN willing to contribute to a training programme for healthcare professionals involved in emergency care, by identifying key considerations for managing the diseases covered by your network?

Yes No Not applicable

Additional comments:

References & Links:

* Could you nominate a member of your ERN who would be willing to serve as a correspondent in a dedicated working group on this topic?

Yes No Not applicable

Additional comments:

References & Links:

* In relation to your ERN's field, would you agree to have the information collected through this survey aggregated into a single document titled "Crisis/Disaster and Rare Diseases", which may be published as a public statement?

- Yes
- No
- Not applicable

Additional comments:

References & Links:

Is there anything else you would like to share about your ERN's experience during crises?

Thank you very much for your contribution
